# Cardiac Regeneration and Tumor Growth—What Do They Have in Common?

**DOI:** 10.3389/fgene.2020.586658

**Published:** 2020-12-09

**Authors:** Severin Dicks, Lonny Jürgensen, Florian Leuschner, David Hassel, Geoffroy Andrieux, Melanie Boerries

**Affiliations:** ^1^Faculty of Medicine, Institute of Medical Bioinformatics and Systems Medicine, Medical Center-University of Freiburg, Albert-Ludwigs-Universität, Freiburg, Germany; ^2^Faculty of Biology, Albert-Ludwigs-Universität, Freiburg, Germany; ^3^Department of Cardiology, Angiology and Pneumology, University of Heidelberg, Heidelberg, Germany; ^4^DZHK (German Centre for Cardiovascular Research), Heidelberg, Germany; ^5^German Cancer Consortium (DKTK), German Cancer Research Center (DKFZ), Freiburg, Germany; ^6^Comprehensive Cancer Center Freiburg (CCCF), Medical Center-University of Freiburg, Freiburg, Germany

**Keywords:** miRNA-sequencing, mRNA-sequencing, zebrafish, heart regeneration, pan cancer

## Abstract

Acute myocardial infarction is a leading cause of death. Unlike most adult mammals, zebrafish have the capability to almost fully regenerate their hearts after injury. In contrast, ischemic damage in adult human and mouse hearts usually results in scar tissue. mRNA-Sequencing (Seq) and miRNA-Seq analyses of heart regeneration in zebrafish over time showed that the process can be divided into three phases: the first phase represents dedifferentiation and proliferation of cells, the second phase is characterized by migration, and in the third phase cell signals indicate heart development and differentiation. The first two phases seem to share major similarities with tumor development and growth. To gain more insight into these similarities between cardiac regeneration and tumor development and growth, we used patient matched tumor normal (“healthy”) RNA-Seq data for several tumor entities from The Cancer Genome Atlas (TCGA). Subsequently, RNA data were processed using the same pipeline for both the zebrafish samples and tumor datasets. Functional analysis showed that multiple Gene Ontology terms (GO terms) are involved in both early stage cardiac regeneration and tumor development/growth across multiple tumor entities. These GO terms are mostly associated with cell cycle processes. Further analysis showed that orthologous genes are the same key players that regulated these changes in both diseases. We also observed that GO terms associated with heart development in the third late phase of cardiac regeneration are downregulated in the tumor entities. Taken together, our analysis illustrates similarities between cardiac remodeling and tumor progression.

## Introduction

Acute myocardial infarction is a leading cause of death (Benjamin et al., 2017). Unlike most adult mammals, zebrafish have the capability to almost fully regenerate their hearts after injury (Poss et al., 2002; Chablais et al., 2011; González-Rosa et al., 2011; Schnabel et al., 2011). In contrast, ischemic damage in adult human and mouse hearts usually results in scar tissue.

In zebrafish, cardiomyocytes can dedifferentiate, proliferate, and invade into the injury site. These processes are considered as an important contribution and prerequisite for heart regeneration. These transcriptional dynamics have been described by numerous time series experiments (Lien et al., 2006; Rodius et al., 2016; Klett et al., 2019). Klett et al. categorized the regeneration process into three phases. The first phase represents cell dedifferentiation and cell proliferation. The second phase is characterized by cell migration. During the third and final phase, cells exhibited cell signals of development and differentiation. Furthermore, it was shown that several miRNAs are crucially involved in the regulation of these processes.

MiRNAs are common ~18–22 nucleotide non-coding RNAs that regulate gene expression at a posttranscriptional level. MiRNAs can bind to mRNAs with matching “seed” sequences (Didiano and Hobert, 2006), and this interaction is associated with transcript destabilization or translation repression (Wang and Li, 2009). Many miRNAs can target multiple mRNAs and many mRNAs can be targeted by multiple miRNAs (Joladarashi et al., 2014).

In this context, miRNAs are known to be an important regulator of tumor proliferation and invasion (Iorio et al., 2005).

Interestingly, the first two phases of heart regeneration of cardiac regeneration are reminiscent of processes of tumor development, metabolism, and growth.

To gain insights into the similarities between the regenerative process of the heart in zebrafish and tumor growth, we have compared these processes in detail. Therefore, we used patient matched paired tumor–normal mRNA and miRNA samples for 17 tumor entities from TCGA. For these data we analyzed the differentially regulated genes, regulated pathways and Hallmarks of Cancer for each tumor entity separately. We compared these results to the dynamic processes of cardiac regeneration on both pathway and gene levels. To investigate the underlying regulation of these processes we also compared the miRNA mRNA interactions.

## Materials and Methods

### Zebrafish Samples

Zebrafish samples were analyzed as described previously in Klett et al. The publicly available zebrafish mRNA and miRNA dataset was used from Klett et al. and is accessible at https://www.ncbi.nlm.nih.gov/sra/PRJNA509429.

### The Cancer Genome Atlas (TCGA) Samples

Paired TCGA mRNA and miRNA Sequencing datasets from malignant tumors were used from TCGA firehose (https://gdac.broadinstitute.org). In total, 17 tumor entities were downloaded and used (Supplementary Table 1, sheet 1). We only considered patients with paired tumor–normal samples. See Supplementary Table 1 for a detailed list of tumor entities and sample numbers.

### Processing of Zebrafish mRNA/miRNA Data

The downloaded FASTQ files were processed with a similar pipeline as described by Klett et al.

Trimmomatic was used for trimming adapters and low-quality reads (Bolger et al., 2014). STAR was used to align to the Ensembl GRCz10 and quantify reads per gene (Dobin et al., 2013). We discarded low-quality samples. For statistical analysis the R/Bioconductor package edgeR was used (Robinson et al., 2010). Data were normalized with TMM (trimmed mean of *M*-values) and tagwise dispersion was calculated (using edgeR). Batch-correction was performed using surrogate variables (Leek and Storey, 2007). We used the same exponential recovery model from Klett et al. for the control group. Differentially expressed genes were defined as FDR <0.01 and absolute log2 fold change (log2FC) > 1 and were calculated with the R/Bioconductor package edgeR.

For the mRNA samples, gene dynamics were soft-clustered into five groups by clustering z-score transformed log2FC between cryoinjured fish and their respective control group, using the R/Bioconductor package mfuzz (Kumar and Futschik, 2007). Five groups were used because it depicts the dynamic of the response. For further analysis we only considered genes within those five clusters (*n* = 4,347) with a cluster association of >70%. For genes within each cluster we performed functional analysis using Fisher's exact test for Gene Ontology biological processes (GO:BP) and Hallmarks of Cancer (Subramanian et al., 2005; Liberzon et al., 2015). A term was considered to be significantly associated with a cluster if FDR was <0.1. For GO:BP we created a graphical network, where all significantly regulated terms for each cluster were considered. To improve visibility and reduce cluster within the network, we discarded terms if they shared more than 98% of their genes with another term in the cluster. Edges were drawn between a term if they shared more than 40% of associated genes with its associated clusters and other terms. This network was dynamically arranged and visualized using Gephi with its algorithm ForceAtlas2 that takes edge weights into account (Bastian et al., 2009; Jacomy et al., 2014).

### Processing of TCGA-Data mRNA/miRNA Data

Each tumor entity of the TCGA data set was analyzed separately based on their count data. In detail, we matched Ensembl IDs with EntrezIDs and if multiple Ensembl IDs were matched to more than one Entrez ID, the one with the largest inter-quartile-range across samples was kept. Genes with <1 count per million in at least 3 samples were filtered out and discarded. We used a paired design for patient matched tumor–normal data to account for heterogeneity between patients. Further procedures and calculations such as TMM normalized, tagwise dispersion, and statistical analysis using edgeR were performed in a similar way as described above for mRNA processing in zebrafish. Differentially expressed genes were defined as FDR <0.01 and absolute log2 fold change (log2FC) > 1 and calculated with the R/Bioconductor package edgeR. If there are <50 up or downregulated mRNAs, the FDR threshold was increased to 0.05.

Paired Gene Set Enrichment Analysis was performed using the R/Bioconductor package gage (Luo et al., 2009) for Gene Ontology biological processes (GO:BP) and Hallmarks of Cancer. Gene sets were considered to be significantly regulated if the FDR was <0.01.

### Processing of Neonatal Mice mRNA Data

We reanalyzed the data from Wang et al. (2019). This dataset is publicly available at https://www.ncbi.nlm.nih.gov/geo/query/acc.cgi?acc=GSE123863. In their study they have, among other things, investigated the transcriptome of the regenerating heart in neonatal mice over 7 days. We only looked at the data from these 1-day-old mice. We took the raw counts from GEO and matched Ensembl IDs with EntrezIDs, and if multiple Ensembl IDs were matched to more than one Entrez ID, the one with the largest inter-quartile-range across samples was kept. Genes with <1 count per million in at least 3 samples were filtered out and discarded. Further procedures and calculations such as TMM normalized, tagwise dispersion, and statistical analysis using edgeR were performed in a similar way as described above for mRNA processing in zebrafish and TCGA. Differentially expressed genes were defined as FDR <0.01 and absolute log2 fold change (log2FC) > 1 and calculated with the R/Bioconductor package edgeR. With the DEGs we performed functional enrichment analysis using Fisher's exact test for Gene Ontology biological processes (GO:BP). A term was considered significant if FDR <0.01.

### Homolog Mapping

For GO and hallmark terms we used the msigdbr R/Bioconductor package that allows the mapping of human genes to their zebrafish homologs and vice versa within the given term. Gene sets were matched by name. The package matches human genes to their zebrafish orthologs within a given term. If a human gene maps to multiple orthologs, we considered all orthologs for the term. Msigdbr also provides a list of references for each mapping of genes across species.

While plotting fold changes within a heatmap, we only considered the first gene, if multiple genes matched to one gene of the other species, for better clarity.

### mRNA-miRNA Interaction

The theoretically predicted miRNA-mRNA interactions of zebrafish were obtained from TargetScan Fish 6.2 (Ulitsky et al., 2012) with a context score <-0.2. For human miRNA-mRNA interactions we used conserved sites with a context score <-0.2 from TargetScan Human 7.2 (Agarwal et al., 2015). Second, for each interaction Pearson correlation coefficients were calculated. In the case of zebrafish, we calculated this interaction individually for each cluster and all significantly regulated miRNA. In the case of human samples, we calculated the interaction for each entity separately. In addition, we only used patient samples, where paired tumor–normal samples for both mRNA and miRNA were available to account for heterogeneity between patients. For each entity we considered only significantly regulated mRNAs and miRNAs. We separately analyzed down regulated miRNA with up regulated mRNA and vice versa.

Interactions were considered valid if ϱ < −0.4 and FDR < 0.05. MiRNAs were combined with each other if they shared a significant number of mRNA interaction targets (hypergeometric test FDR < 0.01). For each correlation analysis we kept the top 15 miRNA with the most valid mRNA interactions. We performed a Gene Set Enrichment Analysis for mRNA targets of each miRNA using Fisher's test for Gene Ontology biological processes (GO:BP) and Hallmarks of Cancer. Terms were considered to be significantly regulated if *p* < 0.01.

## Results

### Zebrafish Heart Regeneration Depicts a Dynamic Process

Lower organisms such as zebrafish are able to maintain the regenerative capacity of the heart by de- and re-differentiating of mature cells and by re-entering the cell cycle. Similar processes are known in the process of tumor development and growth, and it is worthwhile to find out what similarities these processes have.

To study the similarities between heart regeneration and tumor development/growth we first re-analyzed the data from Klett et al. for the aforementioned purpose. The dataset consists of mRNA and miRNA sequencing that captures the dynamic response to cryoinjury in zebrafish. The cryoinjury of zebrafish hearts mimics myocardial injury. To capture the dynamic transcriptional response after cryoinjury the following 10 timepoints were analyzed for mRNA and miRNA after injury: 1, 4, 7, 14, 21, 30, 45, 60, 120, and 160 days post injury (dpi). Four healthy controls of different ages were used to account for potential age-related transcriptome changes. In addition, a group of sham-operated fish was used as a control for transcriptomic changes caused by the surgery. The age of fish when sacrificed is shown in Supplementary Table 1, sheet 2.

**Figure 1:**
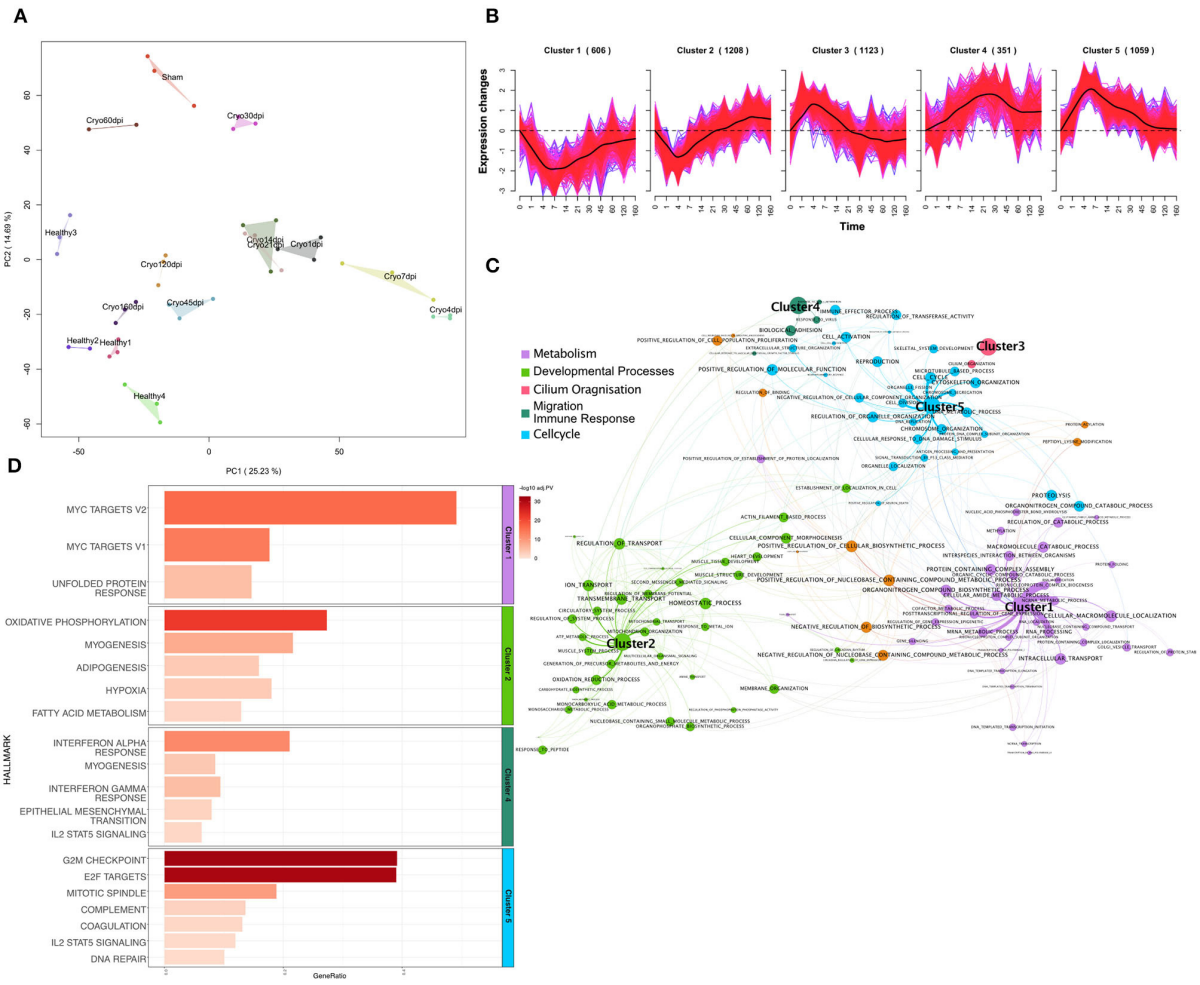
**(A)** Principal component analysis plot of log2-expression (counts per million) of mRNA samples of the zebrafish data. Each color represents a different condition. **(B)** Soft-clustering of log2FC of the zebrafish data over time. Black lines visualize the average dynamics of the clusters. **(C)** Gene Set Enrichment Analysis (Fisher's Test) of genes from every cluster of gene ontology biological processes. Gene sets are connected by an edge if they share 40% of genes. Terms are connected to the clusters based on their p-value. Size of the nodes is anticorrelated to the *p*-value. **(D)** Bar plot of Gene set enrichment analysis (Fisher's Test) of genes from every cluster for Hallmarks of Cancer. The gene-ratio represents the number of regulated genes in the relation to the gene set size. Gene-ratio of genes regulated within each term divided by the total number of genes within the respective term. Color coded bar depicts the –log10 adj. PV (FDR).

These data were pre-processed, normalized, and corrected for unknown batch effects (see Materials and Methods). The principal component analysis (PCA) for the mRNA samples shows a clustering for each timepoint and condition (Figure 1A). The early timepoints (1 dpi to 30 dpi) cluster together away from the later timepoints and healthy controls according to the first principle component. The later timepoints cluster together with the healthy controls. The PCA emphasizes the dynamic changes over time, and it seems that the regenerated hearts at a later time are more similar to the healthy ones than the earlier timepoints, immediately after the injury. All the sham operated samples cluster together in the upper part of the PCA.

Klett et al. used time-dependent weights modeled to describe the influence of sham-operated and healthy fish for the control group. This dynamic control forces the exponential decrease of sham influence over time. We obtained differentially expressed genes (DEGs, FDR < 0.01, and |log2FC|>1) for each time point between the cryoinjured fish and the modeled control.

In accordance with Klett et al. the strongest response peak of DEGs was also captured at 4 dpi and 7 dpi. We could also observe the rapid decline in DEGs for time points after 14 dpi (Supplementary Figure 1).

In the next step, we performed a soft clustering method to gain insight into the dynamic response of cardiac regeneration. Since soft-clustering has a better noise-robustness when compared to hard clustering methods, we used it on z-score transformed log2-fold expression changes of DEGs in at least one timepoint. We could assign 4,347 DEGs (FDR < 0.01 and |log2FC|>1) to five Clusters (Figure 1B), using a cluster association threshold of 70%. The 606 genes in Cluster 1 were downregulated over time, with the strongest downregulation occurring between 4 and 14 dpi. Cluster 2 includes 1208 genes. These genes were downregulated from 1 to 21 dpi, with a downregulated peak at 4 dpi, and after 21 dpi those genes start to be upregulated. Cluster 3 consists of 1,123 genes that are initially upregulated, peaking at 4 dpi, and after 21 dpi we can observe downregulation of these genes. Cluster 4 includes 351 genes that are upregulated over time and shows a broader peak around 21 dpi. Cluster 5 consists of 1,059 genes. The upregulation of those genes peaks at 4 to 7 dpi. The latter then decreases and falls again to the initial value on 120 dpi. For the genes within each cluster we performed a Gene Set Enrichment Analysis (Fisher's exact test) for Gene Ontology Biological Processes (GO:BP) (Figure 1C, materials and methods) and Hallmarks of Cancer (Figure 1D). The genes within Clusters 1 are significantly enriched (FDR < 0.1) for metabolic processes, especially mRNA metabolism. We could also observe the significant downregulation of the hallmarks for myc-targets and unfolded protein response. The genes in Cluster 2, which show an upregulation around 21 dpi, depict a significant enrichment for muscle tissue development and heart development and thus points to a possible starting point of heart regeneration. We further observed enrichment for cell membrane processes and ion transport for this cluster. Accordingly, the top three hallmarks associated with this cluster are oxidative phosphorylation, myogenesis, and adipogenesis. For Cluster 3 we observed an impact on cilium organization for GO:BP but were not able to observe any significant enrichment for hallmark terms. Cluster 4, which contains upregulated genes over time, showed significant enrichment for biological adhesion and immune related GO terms. Additionally, we observed immune related hallmarks and the hallmark for epithelial mesenchymal transition (EMT) for this cluster. In Cluster 5, which shows a peak for upregulated genes between 4 and 7 dpi, we observed GO terms enriched for cell cycle and other processes associated with cell cycle and proliferation processes like DNA replication and cell division. Accordingly, the hallmarks indicate a significant enrichment for G2M checkpoint and E2F targets for this cluster, which also indicates proliferation. Cluster 5 is also characterized by the early immune response. We observed terms associated with macrophage activation and immune effector processes (Supplementary Table 4, sheet 5). Furthermore, we also observed the beginning of EMT (Supplementary Table 5, sheet 5). Interestingly, the functional analysis of the dynamic clusters over time revealed important processes like cell division, immune response, or EMT, which are directly related to tumor development and growth.

### Analysis of Different Tumor Entities

**Figure 2:**
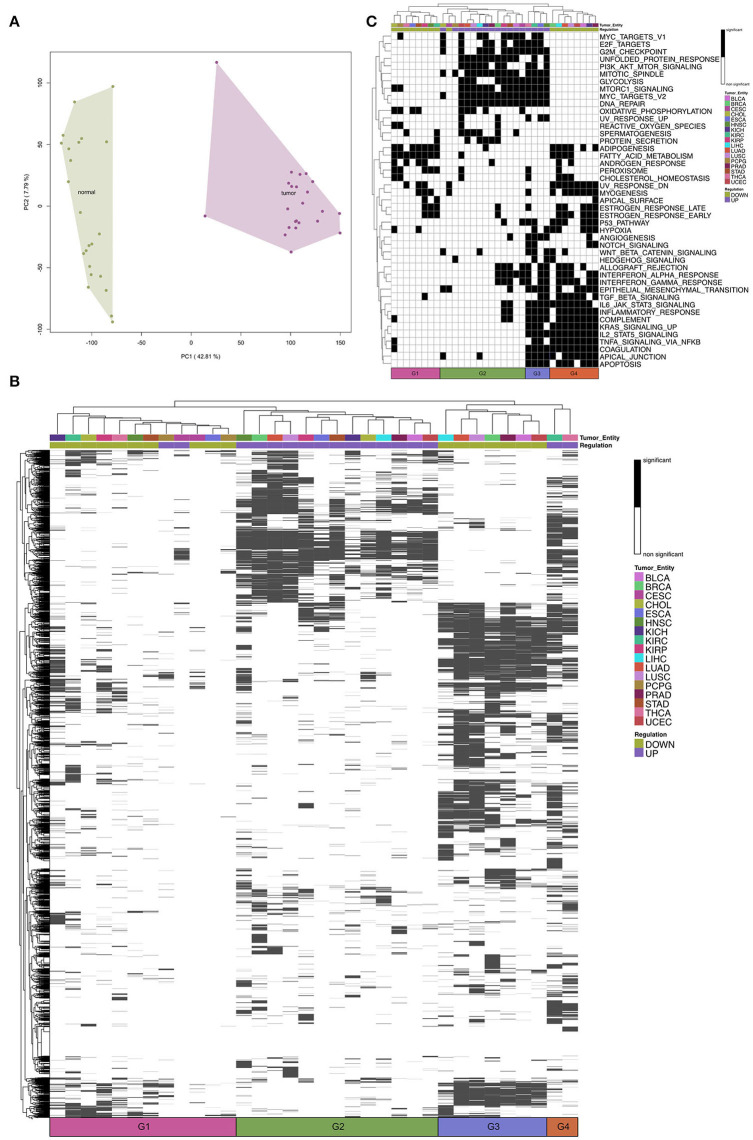
**(A)** A representative Principal Component Analysis of log2-expression (counts per million) of mRNA samples from The Cancer Genome Atlas Kidney Chromophobe (TCGA-KICH) cancer data set. Colors represent the conditions: tumor and normal. **(B)** Heatmap of Gene Set Enrichment Analysis of Gene Ontology biological processes for the tumor entities. Tumor entities and gene sets were hierarchically clustered based on Euclidean distance. The color bar represents whether the given term is significantly regulated. The black and white coded bar represents whether the given term is significantly regulated or not; the color coded bars depict the different tumor entities with the given abbreviation (see Supplementary Table 1) and the up- and down-regulated terms. **(C)** Heatmap of Gene Set Enrichment Analysis of Hallmarks of Cancer for the tumor entities. Tumor entities and gene sets were hierarchically clustered based on Euclidean distance. The black and white coded bar represents whether the given term is significantly regulated or not; the color coded bars depict the different tumor entities with the given abbreviation (see Supplementary Table 1) and the up- and down-regulated terms.

To figure out similarities between cardiac regeneration and tumor development/growth we analyzed 17 tumor entities from TCGA database. For each tumor entity the process of analysis was identical (see Methods). After normalization the PCA revealed a clear separation of tumor samples and the corresponding normal tissue controls (e.g., Figure 2A). We obtained DEGs for each tumor entity in a paired analysis for tumor vs. normal (Supplementary Figure 2). For each entity we performed Gene Set Enrichment Analysis with gage for GO:BP and Hallmarks of Cancer. The comparison of the tumor entities revealed four groups based on hierarchical clustering (Figure 2B, G1–G4). The significantly upregulated terms (FDR < 0.01) within most tumor entities create Group 2. In this group shared terms are mainly involved in cell cycle and metabolism (Supplementary Table 2).

Downregulated GO:BP terms could be divided into two groups (Group 1 and Group 3). Group 3 shares significantly downregulated terms, many involved in development (Supplementary Table 2). Group 1 has a low number of regulated GO terms compared to the other entities. This is correlated to the lower number of samples and the resulting decrease in statistical power.

THCA (Thyroid Carcinoma) and KIRC (Kidney Renal Clear Cell Carcinoma) differ from the other entities based on their significantly upregulated GO terms (Group 4). Although both entities still share the same upregulated terms with the other entities, they also share terms that are in contrast significantly downregulated in the other entities.

When we compare the regulation of Hallmarks of Cancer (Figure 2C) for the tumor entities, we can still see four main groups based on hierarchical clustering. The upregulated hallmarks are clustered within Group 2 and Group 3. We can see that Group 3 (THCA, KIRC, ESCA, and HNSC) shows upregulated hallmarks that are downregulated in the other tumor entities (Group 4). These hallmarks include immunological processes, like interferon response and migratory processes. Group 1 has a low number of downregulated hallmarks compared to the other entities.

Taken together, we observe that most tumor entities share upregulated GO:BP terms and hallmarks involved in cell proliferation and for some tumor entities a downregulation of developmental processes.

### Zebrafish Heart Regeneration vs. Human Tumor Development

For the comparison of the dynamic clusters obtained from zebrafish heart regeneration with the different tumor entities, we first used the significantly regulated GO:BP terms from the zebrafish clusters to evaluate possible common similarities with different tumor entities.

**Figure 3:**
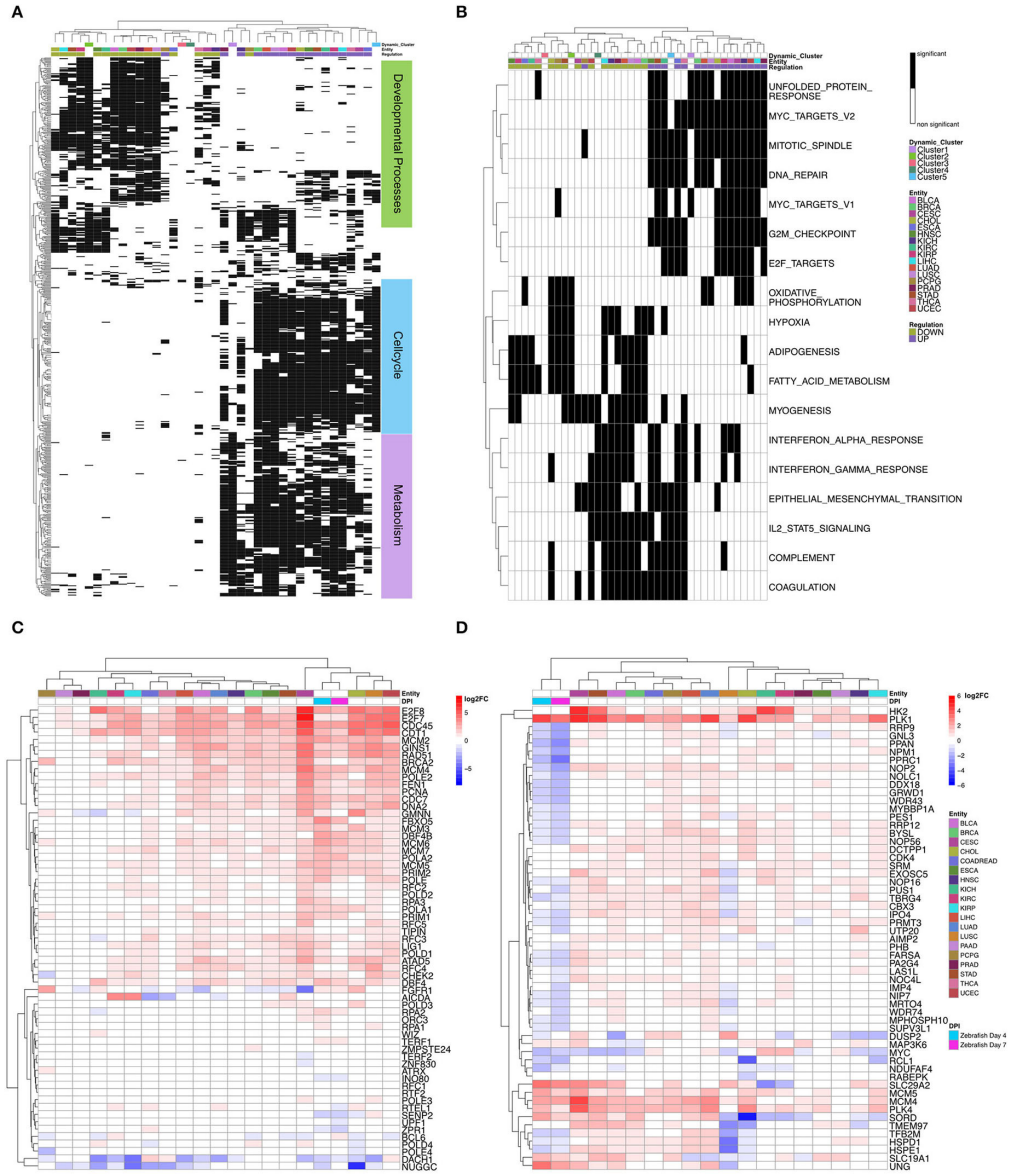
**(A)** Heatmap of the significantly regulated Gene Ontology biological processes terms in the dynamic clusters compared to tumor entities. Dynamic clusters and tumor entities were hierarchically clustered based on their significantly regulated gene sets. The black and white coded bar represents whether the given term is significantly regulated or not; the color coded bars depict the different tumor entities with the given abbreviation (see Supplementary Table 1), the up- and down-regulated terms and the dynamic zebrafish clusters. **(B)** Heatmap of the significantly regulated gene sets Hallmarks of Cancer in the dynamic clusters compared with the tumor entities. Dynamic clusters and tumor entities were hierarchically clustered based on their significantly regulated gene sets. The black and white coded bar represents whether the given term is significantly regulated or not; the color coded bars depict the different tumor entities with the given abbreviation (see Supplementary Table 1), the up-and downregulated terms and the dynamic zebrafish clusters. **(C)** Heatmap of differential expression (log2FC) of all genes in gene ontology biological processes term: cell cycle DNA Replication. Zebrafish samples and tumor entities were hierarchically clustered based on their differential gene expression. The color coded bar depicts the log2FC. **(D)** Heatmap of differential expression (log2FC) of all genes in hallmark: myc targets v2. Zebrafish samples and tumor entities were hierarchically clustered based on their differential gene expression. The color coded bars represent the log2FC, the different tumor entities with the given abbreviation (see Supplementary Table 1) and the two zebrafish days post injury (dpi).

In detail, to compare the dynamic clusters of zebrafish heart regeneration with the different tumor entities, we first used the significantly regulated GO:BP terms from the clusters to compare them with the tumor entities to each other. Cluster 5's terms, which are associated with cell cycle, are also upregulated in the different tumor entities (Figure 3A).

The functional terms of Cluster 1, which are mainly associated to metabolism, are similarly upregulated as the terms of Cluster 5 (cell cycle) in the tumor entities; however, these functional metabolic associated terms are downregulated during the regenerative process in zebrafish and we obtained here an anticorrelation for these processes. Terms obtained from Cluster 2 are involved in heart development and are downregulated until 21 dpi and start to be upregulated after this peak. Interestingly, these terms are mainly downregulated in the different tumor entities. Cluster 3 and 4 don't show any overlap with the tumor entities based on GO:BP.

In addition, we looked at the significantly regulated hallmarks within the clusters and compared them to the tumor entities (Figure 3B). Again, we can observe that hallmarks regulated in Clusters 1 and 5 are also associated with upregulated hallmarks in the tumor entities. The hallmarks significantly enriched, like EMT in Cluster 4, belong to the group of hallmarks that can be up- or down-regulated within the tumor entities. Cluster 2's significantly regulated terms were also mainly downregulated in different tumor entities, except hypoxia, which is upregulated in HNSC and KIRC, and myogenesis, which is upregulated in HNSC Head-Neck Squamous Cell Carcinoma and THCA (Thyroid Carcinoma).

To better understand the regulation of the genes behind these GO:BP terms and hallmarks, we look at the expression of each gene within a term (Figure 3C). We were mainly interested in the cell cycle process during the early stages of cardiac regeneration and tumor proliferation. As an example for such a term we chose the “cell cycle DNA replication.” This term was significantly regulated in Cluster 5. To look at the differential expression for each gene within the term, we chose 4 dpi and 7 dpi, because Cluster 5 strongest regulation was at these time points. We matched the homologous genes between zebrafish and human for this term with the msigdbr package (materials and methods). The heatmap reveals that these homologous genes have a similar regulation between tumor entities and zebrafish cardiac regeneration. Upregulated genes in zebrafish are also upregulated in the tumor entities. Downregulated genes like DACH1 are also downregulated in both zebrafish and multiple tumor entities. DACH1 is a known regulator of retina development (Li et al., 2002). The expression of the zebrafish genes groups within the tumor samples with high expression of the involved genes. The genes involved in proliferation are similarly regulated between the tumor entities and zebrafish. Furthermore, we have also studied the opposite expression of genes between zebrafish and tumor, for example, for the myc targets v2 hallmark (Figure 3D). Most genes within this term are downregulated in zebrafish but upregulated in the different tumor entities. However, there are also few genes like PLK1 and MCM4 that are upregulated in both zebrafish and different tumor entities. PLK1 is known to be an important regulator of heart regeneration in zebrafish and essential for the proliferation of cardiomyocytes (Jopling et al., 2010). Autosomal recessive mutations in MCM4 lead to Immunodeficiency 54 (Gineau et al., 2012).

In an additional analysis, we split the 11 tumor entities with the most patients into early (Stage I and II) and late (Stage III and IV) stages. We compared early and late stages against normal tissue separately for each entity. We performed the functional enrichment analysis GO:BP terms of Cluster 1 (metabolism) and Cluster 5 (cell cycle) with early and late stages of the tumor entities (Supplementary Figure 3A). Most tumor entities' early and late stages cluster together. Only HNSC and BLCA don't cluster together. We can observe, for a given entity, early and late stages are similar.

Furthermore, we compared the scaled –log10 FDR of the enriched GO:BP terms for each stage and each entity. We scaled the –log10 FDR from 0 to 1. For most entities, this results in a linear relationship between early and late stage tumors (Supplementary Figure 3B). For BRCA we can see that in late stage tumors chromosome organization processes are highly upregulated compared to early stage tumors.

### Zebrafish Heart Regeneration vs. Mouse

**Figure 4:**
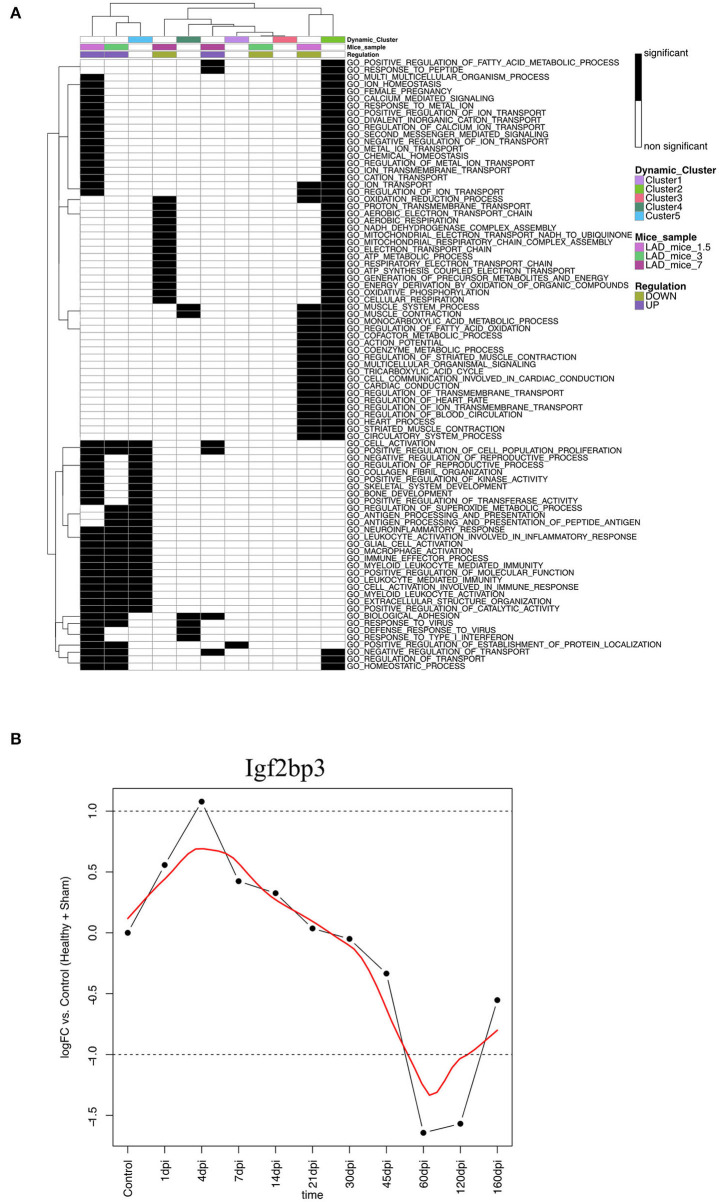
**(A)** Heatmap of the Gene Ontology biological processes terms that are significantly regulated in the zebrafish dynamic clusters and the neonatal mice samples. Dynamic clusters and neonatal mice samples were hierarchically clustered based on their significantly regulated gene sets. The black and white coded bar represents whether the given term is significantly regulated or not. The color coded bars depict the different mice samples, the up- and down-regulated terms and the dynamic zebrafish clusters. **(B)** Differential expression (log2FC) of the gene Igf2bp3 in the zebrafish time series. The red line describes the trend of the log2FC and the black line is a straight connection between time points. The dotted line represents the cut off for significant differential expression.

To validate our findings, we compared the zebrafish dynamic clusters with the early response of heart regeneration in neonatal mice. Therefore, we reanalyzed the data of Wang et al. with our analysis pipeline as described in the method section Processing of TCGA-Data mRNA/miRNA data (Wang et al., 2019). Wang et al. investigated the regenerative potential of neonatal mice after being subjected to the permanent ligation of the left anterior descending (LAD) artery or sham surgery over a short time series (1.5, 4, and 7 dpi) for 1 and 8 days old mice. We only looked at the regenerating samples of 1 day old mice when we compared the functional enriched GO:BP terms with the terms regulated in the zebrafish dynamic clusters. We observed only a small overlap between mice and the dynamic zebrafish clusters (Figure 4A). We see that some early immune related terms in Cluster 5 are shared with Day 1.5 and Day 3 in the neonatal mice, e.g., macrophage activation and immune effector processes. We can also observe that some downregulated terms associated with heart processes on Day 1.5 in neonatal mice are shared with Cluster 2, e.g., regulation of heart rate. A difference is that some metabolic terms upregulated on Day 1.5 in neonatal mice are downregulated during the early stages of heart regeneration in zebrafish, e.g., positive regulation of fatty acid metabolic process. During the regenerative process in mice, we didn't observe upregulated cell cycle related terms. Wang et al. proposed that the lack of upregulated cell cycle terms is caused by high cell cycle activity in newborn mice. Wang et al. pointed out two genes that are distinct for regenerating hearts Igf2bp3 and Ccl24. In our analysis we found that Igf2bp3 is upregulated at early timepoints (Figure 4B). However, the zebrafish ortholog of Ccl24 has been discarded from our analysis due to low counts.

### Comparison miRNA of Tumor and Zebrafish

Klett et al. showed that miRNAs play an important role for regulating heart regeneration. Moreover, miRNAs are also known to be key regulators of tumor development and growth (Iorio et al., 2005). Therefore, we wanted to investigate if the regulation of mRNA by miRNA is comparable between heart regeneration and the tumor entities. We compared the enriched GO:Terms and hallmarks of genes anticorrelated to miRNAs since the interspecies mapping of miRNAs is unreliable.

To identify the interactions during zebrafish heart regeneration, we correlated the mRNAs within each cluster and significantly regulated miRNAs (see materials and methods). For each tumor entity we correlated significantly up- and down-regulated mRNAs with significantly down- and up-regulated miRNAs, respectively. After combining miRNAs with similar mRNA targets, we took the top 15 miRNAs with the most valid mRNA targets (ϱ < −0.4 and FDR < 0.05) for each correlation analysis. We performed Gene Set Enrichment Analysis for mRNA targets for GO:BP and Hallmarks of Cancer.

**Figure 5:**
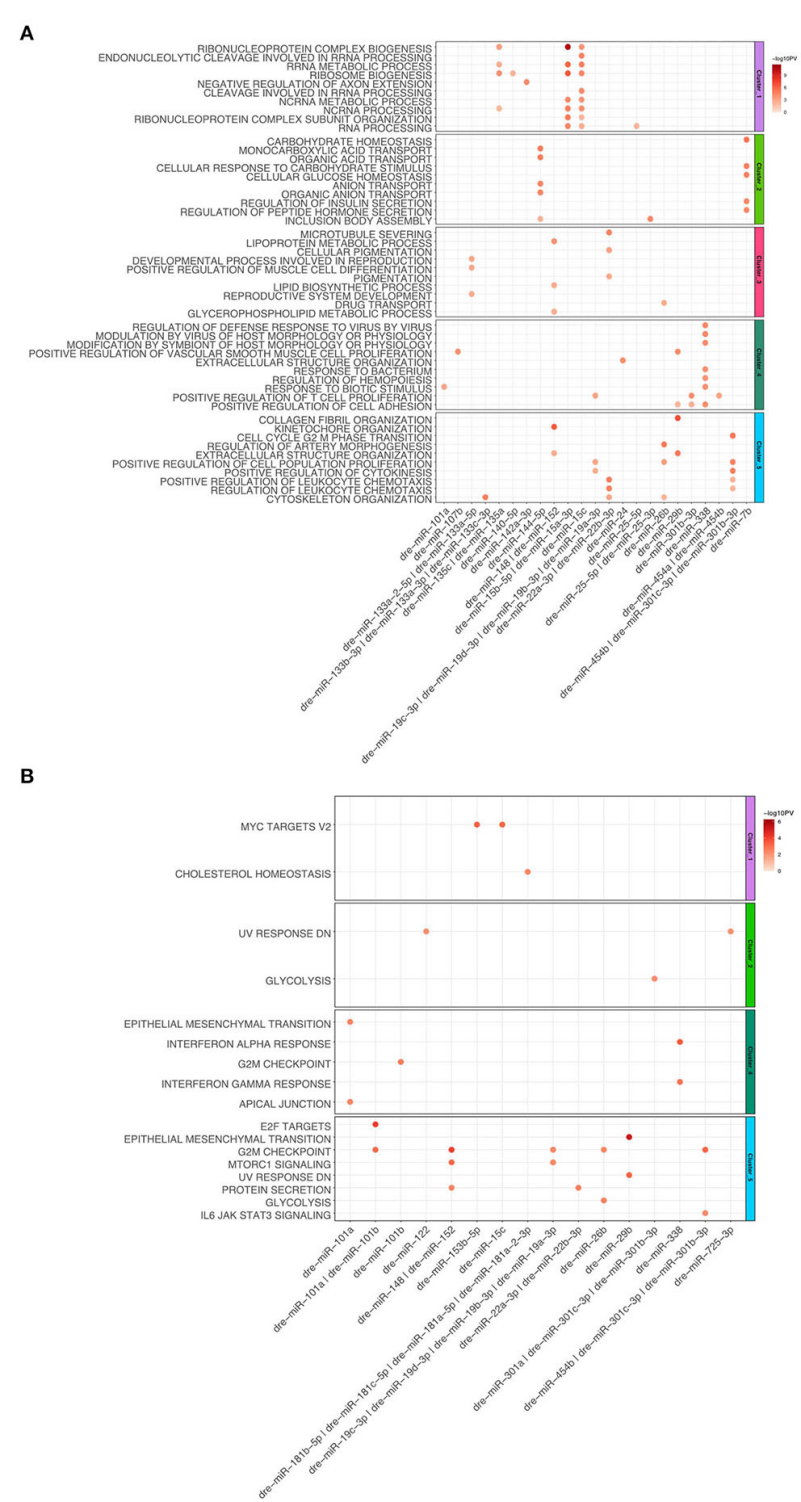
**(A)** Dot plot of Gene Set Enrichment analysis of mRNAs from every cluster regulated by miRNA for Gene Ontology biological processes. For each cluster, only the top 10 terms were shown. The color coded bar depicts the –log10 PV of each term. **(B)** Dot plot of Gene Set Enrichment Analysis of mRNAs regulated by miRNA for every cluster for Hallmarks of Cancer. The color coded bar represents the –log10 PV of each term.

For the heart regeneration clusters we found that mRNA regulated by miRNA are enriched for GO:Terms and hallmarks, which have a similar function to ones described above (Figures 5A,B). Upregulated miRNAs such as dre-miR-15a, b, c anticorrelate to the downregulated mRNA targets in Cluster 1, which are involved in mRNA metabolism. Additionally, the above-mentioned miRNAs are also anticorrelated for genes belonging to the hallmark c-myc targets v2. For Cluster 2 we observed that mRNA anticorrelating to significantly regulated miRNAs (dre-miR-7b and dre-miR-144-5p) were enriched for carbohydrate and lipid metabolism as well as ion transport. Cluster 3 terms are enriched in microtubule processes. MiRNAs anticorrelated with genes from Cluster 3 are dre-miR-133a-5p, dre-miR-133a-2-5p, dre-miR-148, dre-miR-152, dre-miR-22a-3p, and dre-miR-22b-3p. For Cluster 4 we observed that upregulated mRNAs, which correlate to significantly downregulated miRNAs, are enriched for immune processes and cell migration. Both GO:BP terms and hallmarks associated with immune response are linked to dre-miR-338. Genes associated with the hallmark for EMT are anticorrelated with the miRNA dre-miR-101a. For Cluster 5 we observed that mRNA interaction targets of miRNA are mainly involved in cell cycle processes. The top 10 interactions in Cluster 5 are anticorrelated to numerous miRNAs. Dre-miR-101a and dre-miR-101b are anticorrelated with genes that are enriched for hallmarks E2F targets and G2M checkpoint. G2M checkpoint is also linked to dre-miR-148, dre-miR-152, dre-miR-454b, dre-miR-301c-.3p, and dre-miR-301b-3p. Taken together, multiple genes within GO terms and hallmarks are regulated by miRNAs for the zebrafish dynamic clusters during heart regeneration.

**Figure 6:**
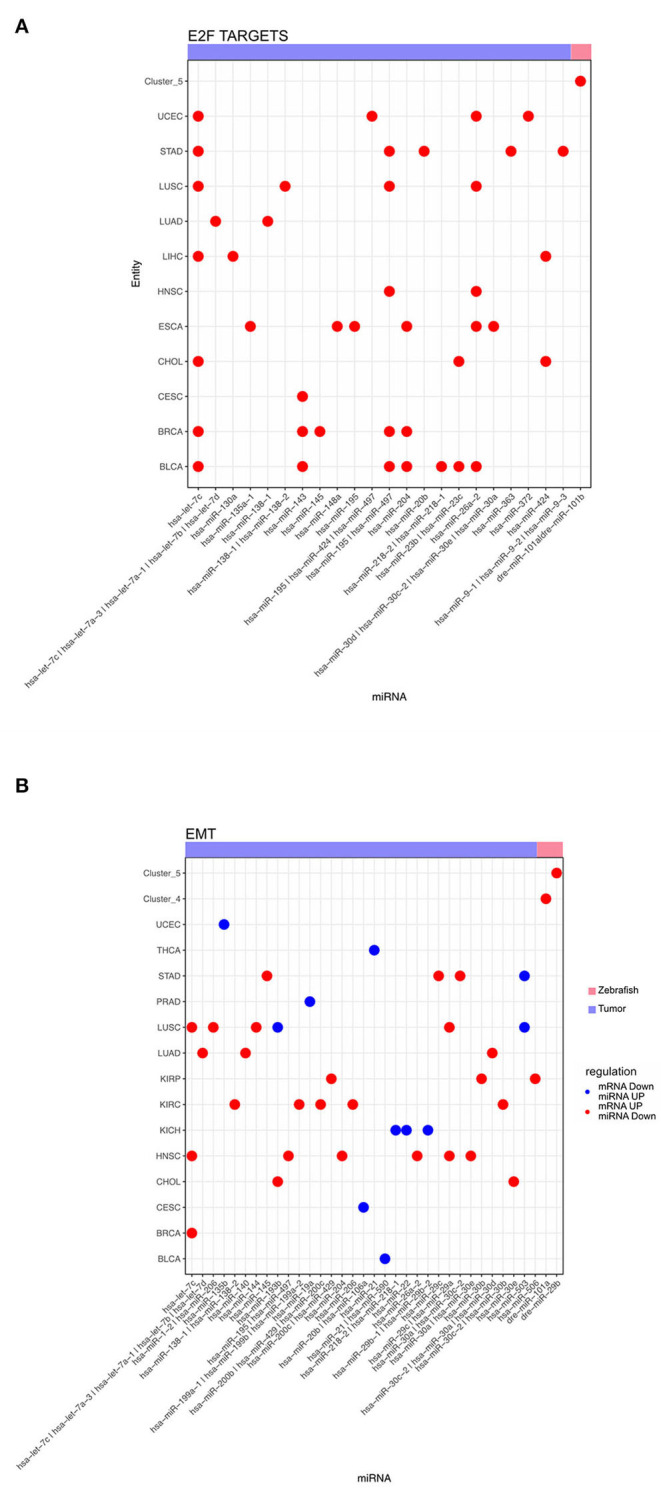
Dot plot of miRNA that significantly regulates the hallmarks **(A)** E2F targets and **(B)** epithelial mesenchymal transition for zebrafish dynamic clusters and tumor entities.

Additionally, we analyzed the miRNA, responsible for anticorrelated genes regulating the hallmarks for E2F targets and EMT, across different tumor entities and dynamic zebrafish Clusters. For E2F targets we observed multiple miRNAs predicted regulating this hallmark across several tumor entities (Figure 6A). Some miRNAs, such as has-let-7c, play a role in regulating these E2F targets across multiple entities. However, there are also other miRNAs that are exclusive to a single entity, like has-miR-148 for ESCA. For zebrafish, only dre-miR-101a and dre-miR-101b are regulating this hallmark in Cluster 5. For the hallmark EMT a diverse group of miRNAs is involved in its regulation (Figure 6B). We observed that none of the miRNAs are involved in the regulation of more than 3 entities in contrast to the previous E2F targets (Figure 6A). In some entities like LUSC miRNA are both up- and down-regulated for EMT genes. Furthermore, EMT related genes are regulated by dre-miR-101a in the migratory Cluster 4 and also by dre-miR-29b depending to the cell cycle Cluster 5. Thus, multiple tumor entities share miRNAs that are negatively correlated to mRNAs enriched for both hallmarks.

**Figure 7:**
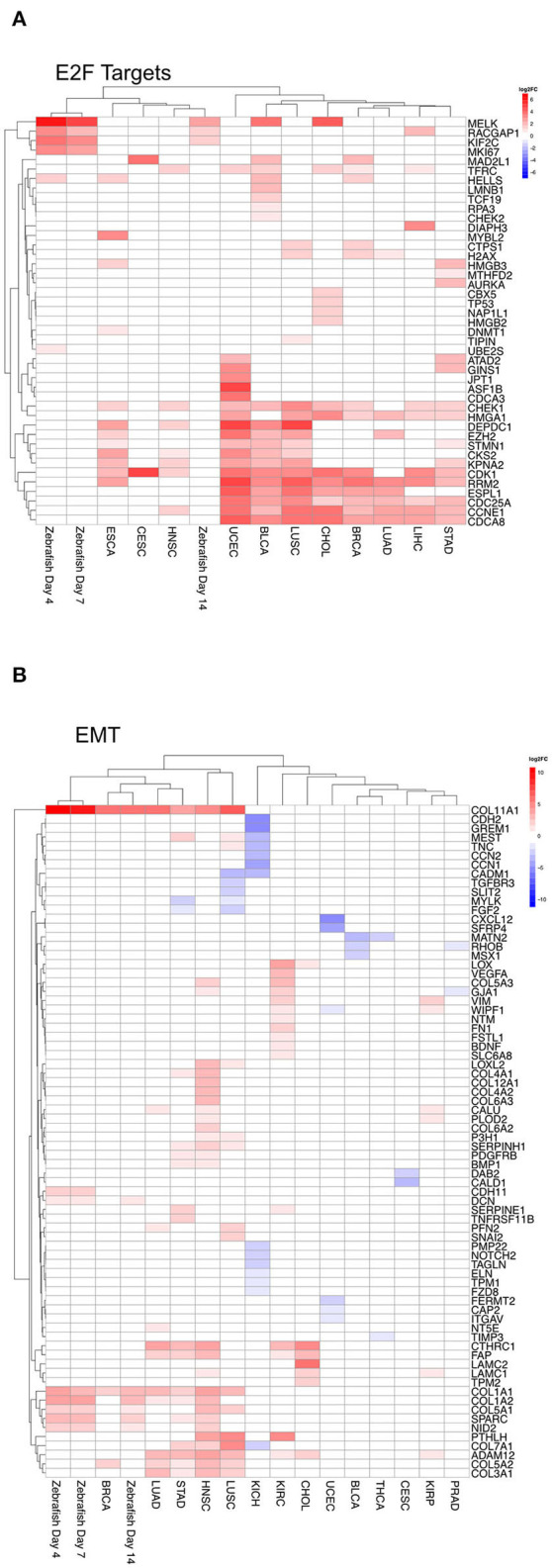
Heatmap of differential gene expression of significantly regulated mRNAs modulated by miRNA in the hallmarks **(A)** E2F targets and **(B)** epithelial mesenchymal transition for zebrafish samples and tumor entities. Dynamic clusters and tumor entities were hierarchically clustered based on their differentially expressed mRNAs. The color coded bar represents the log2FC.

Furthermore, to compare in detail mRNA negatively correlated to the respective miRNA within a given term, we analyzed on the individual gene level for both E2F targets and EMT. For these two hallmarks we compared the differential mRNA expression predicted to be regulated by miRNAs for each tumor entity and the zebrafish samples. For zebrafish, we focused on the following dates: 4, 7, 14 dpi, because they corresponded mainly to both peaks within Clusters 4 and 5. For E2F targets, which plays an important role in cell cycle (Figure 7A), we observed an overlap of genes predicted to be regulated by miRNAs within the tumor entities. In zebrafish, regulated genes are shared with some tumor entities. For the hallmark EMT (Figure 7B) we observed an overlap between tumor entities, where EMT is upregulated as shown in Figure 6B. The genes COL11A1 and COL1A1 are upregulated by miRNAs across five tumor entities (BRCA, LUAD, STAD, HNSC, and LUSC) and zebrafish samples (4, 7, and 14 dpi). Collagen proteins are an important part of the extracellular matrix (Ricard-Blum, 2011).

## Discussion

In this study we compared dynamic transcriptome data from zebrafish heart regeneration with several tumor entities, to figure out if there are similar and even different processes between these data sets.

The time series analysis revealed 5 distinct clusters. Cluster 1 showed downregulation of metabolic and mRNA processes. Cluster 2 is characterized by heart and muscle development. Cluster 3 shows cilium processes. Genes within Cluster 4 are enriched for cell adhesion and EMT and Cluster 5 is mainly involved in cell cycle processes and cell division.

Cluster 5 shares a lot of similar gene sets with multiple tumor entities (Figures 3A,B). These pathways are mainly involved in cell cycle, cell division, DNA damage repair, and DNA replication (Figure 1C). Cluster 5 also shows common Hallmarks of Cancer like E2F targets and G2M checkpoint (Figure 1D). For the term cell cycle DNA replication we can see that they share a similar expression profile (Figure 3C). This leads to the conclusion that the underlying mechanism of cells in heart regeneration as well in tumor growth in proliferation is similar.

Genes of Cluster 1 are involved in metabolic differences and are downregulated in heart regeneration, but they show the opposite behavior in the different tumor entities. The latter is known and an altered metabolism is a prerequisite for tumor growth (Vazquez et al., 2016). Another major difference between zebrafish heart regeneration and tumor growth is that the hallmarks for c-myc targets are downregulated during the regeneration, while these targets are upregulated in most tumors (Figures 3B,D). Myc targets are known tumor drivers (Ben-Yosef et al., 1998) that play a major role in tumor development and growth. Interestingly, since they are downregulated during heart regeneration, this could be a possible explanation for how zebrafish could control the process of cell proliferation, but this will need further analysis in the future.

Cluster 4, responsible for processes that are involved in cell migration, shares some similarities with the different tumor entities. This cluster depicts the well-known process of epithelial mesenchymal transition (EMT) that is a prerequisite for a tumor cell to migrate and metastasize and plays also key roles in embryogenesis and wound healing (Chaffer et al., 2016). From this analysis it can be concluded that zebrafish cells during heart regeneration use migration mechanisms similar to those used in cancer.

Cluster 2 is involved in developmental processes. GO:BP terms regulated within this cluster are mainly in the later stage of the regeneration process. Interestingly, almost every term that is enriched in this cluster is downregulated in the tumor entities. It should be noted, however, that in these used tumor data we are only looking at a snapshot, so we are not able to assess the dynamic process of tumor development.

At the mRNA level, we found many similarities when it comes to the processes of proliferation and migration. We were able to show that mechanisms that promote cell proliferation in the early phase of the heart regeneration are similar to those that promote proliferation in the tumor entities. However, we could also show that metabolic processes are downregulated during heart regeneration in contrast to several cancers, and this might be a further explanation how zebrafish can control proliferation during regeneration.

We also compared the early and late stages of the tumor entities with Cluster 1 and Cluster 5. Those Clusters are enriched for terms that are also upregulated in the tumor entities. We were able to show that there is in general no difference in upregulated terms that correspond to the zebrafish clusters in regards to the tumor stage. When we compared the scaled *p*-values directly between early and late stage tumors of the same entity, we saw an almost linear correlation.

When we compared the neonatal mice data with our dynamic clusters, we saw that only a few terms are shared between heart regeneration in mice and zebrafish. However, these processes are involved in the early immune response to the ischemic damage. We also see that heart regulatory processes are downregulated in early stage heart regeneration in both zebrafish and neonatal mice. The lack of cell cycle activity is most likely due to the fact that neonatal mice hearts are still growing and proliferating. Thus, the difference between sham operated and myocardium injured mice is not significant. We also think that neonatal mice and adult zebrafish are difficult to compare due to the difference in the developmental stage.

To elucidate whether the underlying regulation by miRNA of these genes is similar, we compared the interactions between mRNA and miRNAs. We could confirm that the mRNAs in Cluster 1 are targets of miRNAs and that those target mRNAs are enriched for metabolism and the hallmark myc targets. This leads to the conclusion that miRNAs are regulating those terms and its functional processes (Figures 5A,B).

In addition, we observed that target genes of miRNAs in Clusters 4 and 5 are enriched for the hallmark EMT. This enrichment is also consistent with the regulation on the mRNA level. We noticed that in the entities LUSC and STAD these mRNA targets of this hallmark mediated by miRNA are both down- and up-regulated. Furthermore, EMT is regulated by different miRNAs for several tumor entities (Figure 6B), and in this context, it has been shown that multiple miRNAs are involved in regulating EMT (Bullock et al., 2012; Zhang and Ma, 2012). We could identify some previously known miRNAs associated with EMT in the tumor entities like the hs-miR-200 family and hs-miR-30. Some miRNAs we identified were known to be associated with metastasis and invasion in general, like hs-let-7. However, on the mRNA level this diversity is not that pronounced. Some collagen genes are upregulated in five tumor entities with a positive regulation of EMT by miRNAs. These collagen genes are also upregulated by miRNAs in zebrafish.

The mRNAs in Cluster 5 regulated by miRNA are enriched for E2F targets. mRNA targets regulated in the different tumor entities are also enriched for this hallmark. In zebrafish mRNAs are the targets of dre-miR-101a and dre-miR101b. In human, the most prevalent miRNA that targets these genes is hs-let-7c. This miRNA targets 7 of 11 entities. The hs-let-7 miRNA family is known to be regulating proliferation (Yu et al., 2007). In most tumor entities we could also observe that hs-miR-195 was downregulated. In bladder cancer decreased expression of hs-miR-195 is associated with reduced survival (Yang et al., 2019). The genes regulated by these miRNAs show a similar pattern across most tumor entities. In zebrafish these mRNAs targets regulated by miRNAs don't share many similarities with the tumor entities. It seems that even if the outcome of regulation is similar, such as the upregulation of E2F targets, the underlying responsible genes can be different.

Taken together, we observed that the first two phases of heart regeneration in zebrafish shared common processes, such as cell proliferation and migration, to tumor development and growth in human.

## Data Availability Statement

Publicly available datasets were analyzed in this study. This data can be found at: TCGA Firehose: https://gdac.broadinstitute.org; zebrafish heart regeneration data: https://www.ncbi.nlm.nih.gov/sra/PRJNA509429. The mouse dataset is publicly available at https://www.ncbi.nlm.nih.gov/geo/query/acc.cgi?acc=GSE123863.

## Author Contributions

SD, GA, and MB designed the study and wrote the manuscript. SD analyzed the data. GA and MB created the scientific strategy for the project. All authors read, critically revised, and approved the final version of the manuscript.

## Conflict of Interest

The authors declare that the research was conducted in the absence of any commercial or financial relationships that could be construed as a potential conflict of interest.
