# Supplementary material for: Cardiac Regeneration and Tumor Growth—What Do They Have in Common?
Source: Front Genet. 2020 Dec 9;11:586658. doi: 10.3389/fgene.2020.586658 (PMC7756072; doi:10.3389/fgene.2020.586658)
Supplement: Supplementary file 6 [file Data_Sheet_1.PDF]

Supplementary Material

1.1 Supplementary Figures

Supplementary Figure 1.

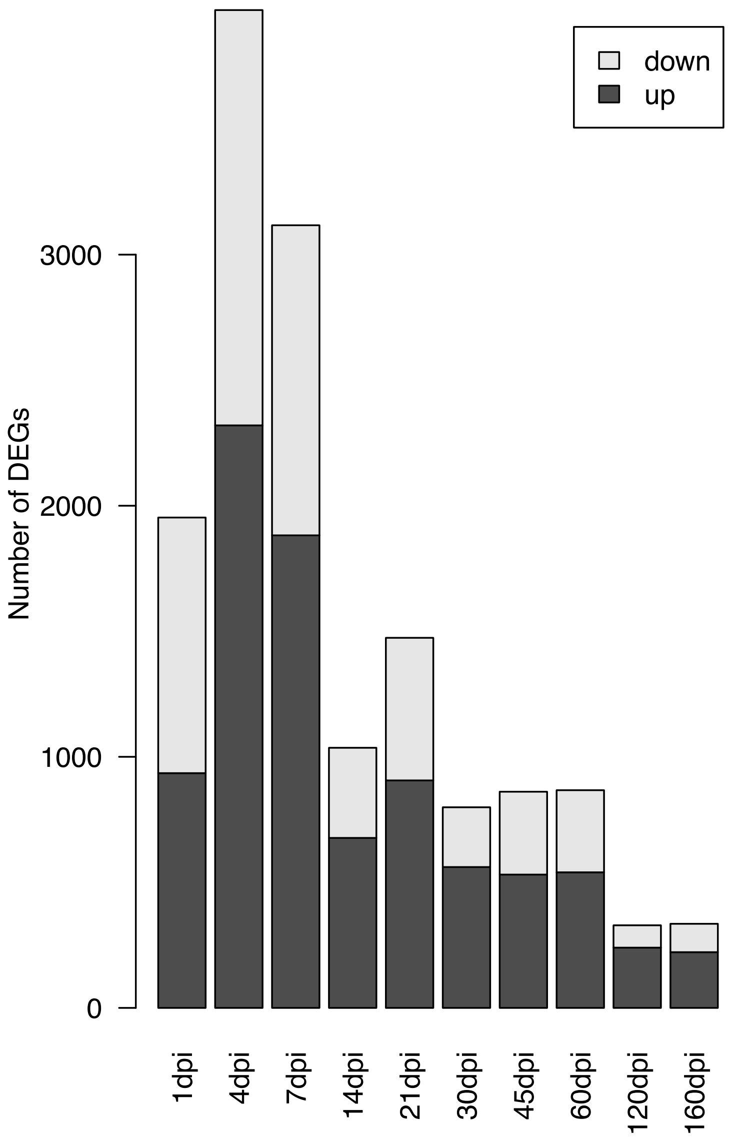

Barplot of numbers of significantly up- and downregulated genes for the zebrafish samples (FDR < 0.01 & |log2FC|>1)

Supplementary Figure 2.

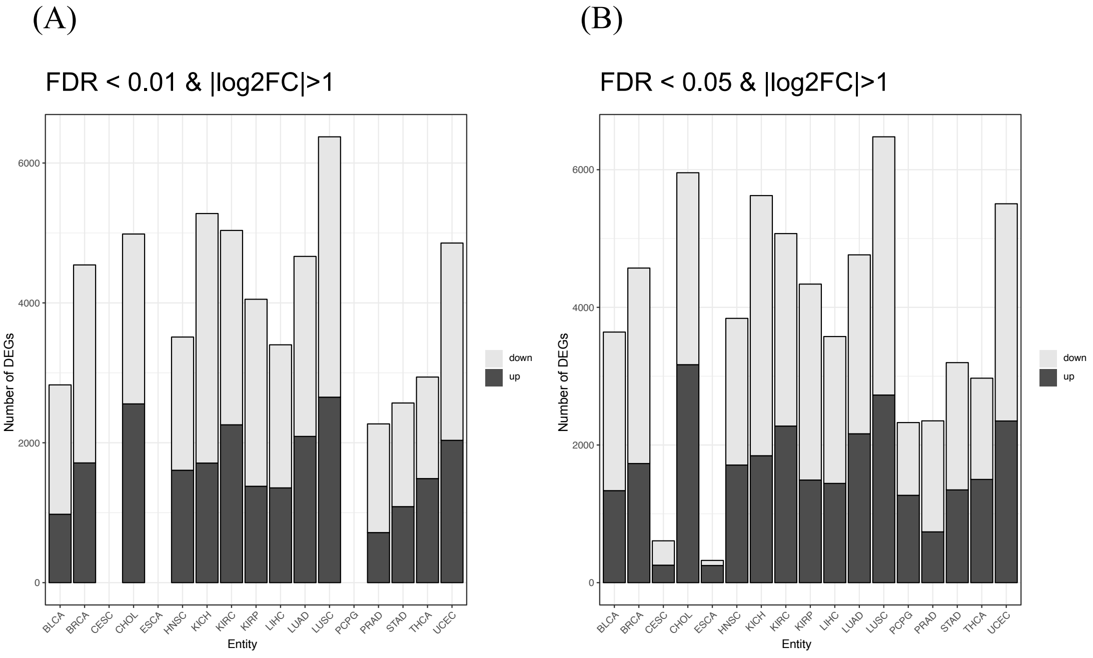

(A) Barplot of numbers of significantly up- and downregulated genes for the tumor entities (FDR < 0.01 & |log2FC|>1)

(B) Barplot of numbers of significantly up- and downregulated genes for the tumor entities (FDR < 0.05 & |log2FC|>1)

Supplementary Figure 3.

(A)

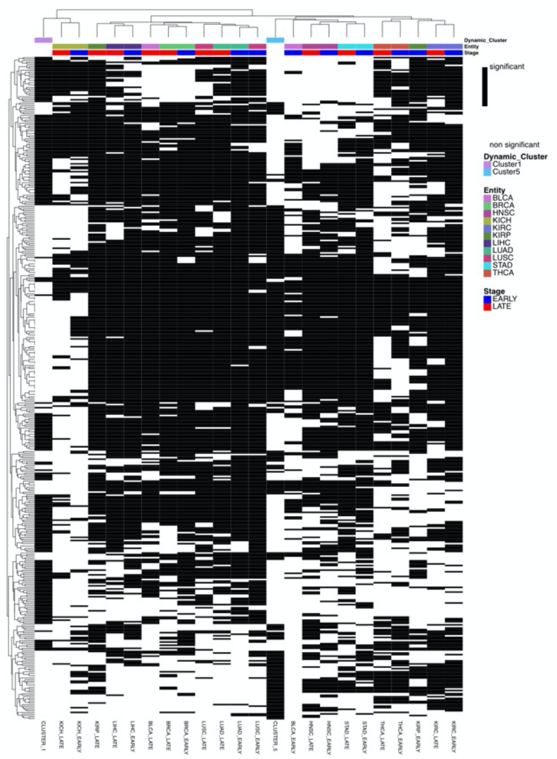

(B)

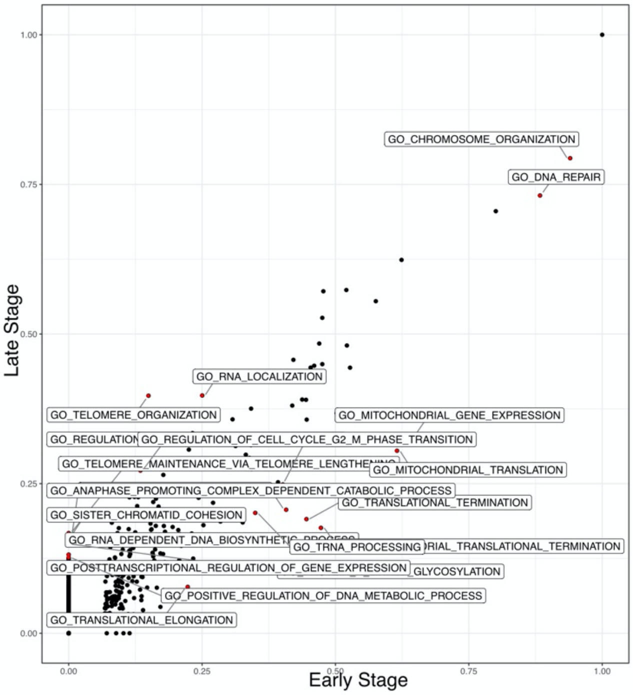

(A) Heatmap of the significantly regulated gene sets Gene Ontology biological processes in the dynamic clusters 1 and 5 compared to the early and late stage tumors for each entity. Dynamic

clusters and tumor stages were hierarchically clustered based on their significantly regulated gene sets. The black and white coded bar represents whether the given term is significantly regulated or not, the color coded bars depicts the different tumor entities with the given abbreviation (see Supplementary Table 1), the tumor stage and the dynamic zebrafish clusters.

(B) Dotplot of the scaled -log10 FDR between 0 and 1 of the enriched GO:BP terms for early and late stage BRCA tumors. Red dots are the top 10 terms with the largest difference between early and late stage.

1.2 Supplementary Tables

Supplementary Table 1

Sheet1: Table with tumor entities abbreviations with full names and number of TCGA patients and sample size for each analysis

Sheet2: Age of zebrafish sample when operated and sacrificed in days.

Supplementary Table 2

Table with FDRs of the Gene Set Enrichment Analysis of Gene Ontology biological process terms up and downregulated for the individual tumor entities. FDRs above 0.1 is set to 1.

Supplementary Table 3

Table with FDRs of the Gene Set Enrichment Analysis of hallmarks up and downregulated for the tumor entities. FDRs above 0.1 is set to 1.

Supplementary Table 4

Table with the results of the functional enrichment for Gene Ontology biological process for each dynamic zebrafish cluster. Entrez IDs and symbols correspond to the genes within the clusters for each term.

Supplementary Table 5

Table with the results of functional enrichment for hallmarks for each dynamic zebrafish cluster. Entrez IDs and symbols correspond to the genes within the clusters for each term.
